# Association between Ultraprocessed Food Intake and Overweight, Obesity, and Malnutrition among Children in Tehran, Iran

**DOI:** 10.1155/2022/8310260

**Published:** 2022-08-24

**Authors:** Elaheh Asgari, Mohammadreza Askari, Nick Bellissimo, Leila Azadbakht

**Affiliations:** ^1^Department of Community Nutrition, School of Nutritional Sciences and Dietetics, Tehran University of Medical Sciences, Tehran 14167-53955, Iran; ^2^School of Nutrition, Ryerson University, Toronto ON M5B-2K3, Canada; ^3^Diabetes Research Center, Endocrinology and Metabolism Clinical Sciences Institute, Tehran University of Medical Sciences, Tehran 14167-53955, Iran

## Abstract

Childhood is a critical period for susceptibility to malnutrition. The consumption of ultraprocessed foods (UPFs) has been increasing among children. The objective of this study was to evaluate the relationship between UPF intake and overweight/obesity and malnutrition in children. 788 children aged 6 years were included in a population-based cross-sectional study in Tehran. A 168-item semiquantitative food frequency questionnaire was used to evaluate dietary intake. UPFs were detected using the NOVA classiﬁcation system. Logistic regression analyses were used, and results were reported as odds ratios (ORs) and 95% confidence interval (CI) of obesity and malnutrition across the tertiles of UPFs adjusted for energy intake, socioeconomic status, and physical activity. The mean weight, height, BMI, and total energy intake of participants were 20.85 ± 2.35 kg, 113.75 ± 2.00 cm, 16.12 + 1.84 kg/m^2^, and 1014.74 ± 259.16 (kcal/d), respectively. There were no significant associations between UPF intake and obesity (OR = 0.97; 95% CI 0.31 to 3.01; P-trend = 0.98), wasting (OR = 0.94; 95% CI 0.30 to 2.87; P-trend = 0.87), overweight/obesity (OR = 0.86; 95% CI 0.59 to 1.25; P-trend = 0.45), underweight/wasting (OR = 0.69; 95% CI 0.40 to 1.17; P-trend = 0.17), marginal-stunting (OR = 1.16; 95% CI 0.71 to 1.89; P-trend = 0.53), or marginal-stunting/overweight/obesity (OR = 1.25; 95% CI 0.62 to 2.54; P-trend = 0.47). There was no evidence of an association between intake of UPFs and risk of overweight, obesity, and malnutrition in children.

## 1. Introduction

Childhood is a critical period for susceptibility to malnutrition [1]. Malnutrition in children is a global burden with economic consequences in developing countries, particularly in Asia [2]. Malnutrition in childhood is associated with approximately half of all child deaths worldwide [3]. As we know, the definition of malnutrition includes both under and overnutrition [4]. Childhood obesity is highly prevalent and can track into adulthood resulting in long-term health consequences [5]. In 2019, it was estimated that 144 million preschool children had stunting, 6.9% had wasting, 2.1% had severe wasting, and approximately 38.3 million children were overweight globally [6]. In Iran, 3.6% of children aged 6 years were obese and 8.4% were overweight [7]. In Iranian preschool children, the rates of being underweight, stunting, and wasting were 7.5%, 12.5%, and 4.4%, respectively [8].

Several factors such as family, environment [9], dietary habits [10], and infectious diseases contribute to obesity [11] and malnutrition [12]. Dietary habits are a modifiable risk factor for obesity and malnutrition. Recent changes to food processing have affected global food systems. Sophisticated food processing methods have resulted in a modified structure and content of the nutrient in a certain food. Ultraprocessed foods (UPFs) which undergo the highest level of processing [13] are defined as multi-component manufacturing formulations including salty snacks, readily prepared frozen meals, sugar-sweetened beverages (SSBs), ice cream, candy, cookies, industrial bread, and breakfast cereal [14, 15]. The percentage of UPF intake has a positive association with caloric density of the diet and dietary intakes of total fat, saturated fat, total carbohydrates, and free sugar and an inverse association with protein and fiber intake [16, 17]. Due to delayed economic growth in developing countries, a smaller percentage of UPF intake is observed [18]. UPF consumption is highest in Australia, North America, Europe, and Latin America, but is increasing rapidly in Asia, the Middle East, and Africa [19]. A systematic review concluded that high UPF intake has a positive association with the risk of all-cause mortality, and many non-communicable diseases such as cerebrovascular diseases, hypertension, metabolic syndrome, overweight, and obesity in adults [20].

An epidemiological study found that UPF consumption may be associated with a higher risk of obesity, overweight, and stunting in low and middle-income countries [21], while another study found an association between UPF consumption and higher waist circumference in children [22]. There has been a rapid increase in the consumption of UPFs, which can decrease dietary quality [22]. To the best of our knowledge, no study has evaluated the association between UPF intake and overweight, obesity, and malnutrition in preschool-aged children in the Middle East. Therefore, the objective of this study was to evaluate this relationship.

## 2. Materials and Methods

### 2.1. Participants

BMI was selected as the primary outcome to estimate the sample size requirement. The value of “*d*” was also considered equal to 0.015% of the average. The “*d*” is the rate of changes in the amount of BMI between groups based on the researcher's opinion. BMI (mean) = 25.9 SD = 3.62 *d* = 3% ^*∗*^ 22 = 0.66 *Z* (1-*α*/2) 2 × SD2/d2. Due to a possible decline in the number of participants, 788 children aged 6 years participated in this cross-sectional study. Children were recruited from healthcare centers in Tehran from 2017 to 2018 using a random cluster sampling method. In this study, we randomly selected regions across all of the regions of Tehran. Originally, all regions were included in the sampling method to incorporate regions with varying levels of socioeconomic status. Healthcare centers were then randomly chosen from selective regions. Records for children aged 6 years were obtained from each healthcare center and the children were randomly selected according to a computer-based random sequencing program. Inclusion criteria were: children aged 6 years, consent provided by the child's parents, and the absence of diseases. The Ethics Commission of Tehran University of Medical Sciences approved this study (IR.TUMS.VCR.REC. 99-2-212-49976).

### 2.2. Dietary Assessment and UPFs Calculation

A validated and reliable 168-item semi-quantitative food frequency questionnaire (FFQ) [23] was used to collect dietary intakes over the past year. For each child, the mother was interviewed to determine the frequency of consumption for each food obtained on a daily, weekly, monthly, and annual basis. We determined the amount of food using standardized measurement tools, which were converted to grams. Energy intake, nutrient intake, and UPFs were determined using the NUTRITIONIST 4 software. To evaluate the dietary intake of UPFs in this study, participants were categorized into tertiles.

According to the NOVA (a name, not an acronym) classiﬁcation system [14], UPFs were determined. This classification system divides foods into four different groups [13, 14, 22] as presented in Supplementary Table 1. The UPFs group includes biscuits, cakes and pastries, ice cream, jams (preserves), chocolates, confectionery, cereal bars, breakfast cereals with added sugar, chips, crisps, sauces, savory and sweet snack products, sweetened fruit and milk drinks, sweetened and non-caloric cola, and other soft drinks; pre-prepared pizza and pasta, meat, poultry, fish and vegetable dishes; chicken nuggets, hot dogs, sausages, burgers, fish sticks; canned or dehydrated soups and noodles; and infant formulas, follow-on milk, and baby food [24]. These received the highest level of processing and were produced as multi-component industrialized formulations [13]. According to a review of the FFQ, 33 items were included in the UPFs list. Daily intake of UPFs in grams was obtained by adding up their daily consumption. Standardization by calories of UPF was completed, and according to a study conducted in Iran with a few appropriate changes [25], we classified UPFs into seven separate food groups. These seven food groups and their components are available in Supplementary Table 2.

### 2.3. Anthropometric Definition and Assessment

Weight, height, and mid-upper arm circumference (MUAC) were measured. Weight was measured using an electronic scale (Seca 753E; Germany) with an accuracy of 100 g while participants were wearing light clothing and no shoes. Height was measured with 0.1 cm precision in a standing position while the participant's shoulders were in a relaxed position and were barefoot. Body Mass Index (BMI) was obtained by dividing weight (kg) by height (meters squared). BMI for age and sex was classified into: obese (BMI-for-age ≥2 SD), overweight (BMI-for-age ≥1 SD), normal (BMI-forage ≥ −1 SD and <1 SD), underweight (BMI-for-age < −1 SD), and wasting (BMI-for-age < −2 SD) [26], and height-for-age *Z* (HAZ) scores were −2˂ HAZ ≤ −1 SD defined as marginal-stunted [27]. MUAC was measured by determining the mid-point of the arm between the top of the shoulder and the apex of the elbow, followed by measuring the circumference at the mid-point using a tape measure.

### 2.4. Physical Activity (PA)

PA level was evaluated using the international physical activity questionnaire (IPAQ) for children collected by a trained interviewer [28]. Based on the frequency and duration of PA, activities were categorized into light, moderate, high, and very high-intensity over the past year, according to the usual daily activities list. PA levels were reported as metabolic equivalent hours per week.

### 2.5. Socioeconomic Factors

Socioeconomic status (SES) was determined using a validated and reliable scale developed for assessing socioeconomic status [29]. SES consisted of educational level, income, number of family members, house ownership or tenancy, area of housing, car ownership, number of cars, and type and number of bedrooms in the house.

### 2.6. Statistical Methods

Statistical analyses were performed using the SPSS software (version 23). Statistical significance was considered as *P* < 0.05. The Kolmogorov-Smirnov test was used to survey the normality of the distribution for quantitative variables. In the UPFs tertile, continuous variables were reported as mean and standard deviation (SD). Covariance analysis was used to compare dietary intakes across the tertiles of UPFs adjusted for daily energy intakes. By variance analysis was performed to compare the means of quantitative variables across the UPFs tertiles. To assess the risk of malnutrition, simple logistic regression and multivariate logistic regression models were used. The multivariate model was adjusted for energy intake, socioeconomic status, and physical activity. The stepwise selection method was used to select the independent variables.

## 3. Results

This cross-sectional study was conducted on 6-year-old children. The percent contribution of each UPF group to the total daily caloric intake was as follows: Group 1, 0.29%; Group 2, 11.36%; Group 3, 22.10%; Group 4, 5.30%; Group 5, 6.49%; Group 6, 2.15%; and Group 7, 9.59%. The percent contribution of total UPF to the total daily caloric intake was 57.31%. The mean and SD for weight, height, BMI, and total-energy-intake of participants were 20.85 ± 2.35 kg, 113.75 ± 2.00 cm, 16.12 + 1.84 kg/m^2^, and 1014.74 ± 259.16(kcal/d), respectively. The prevalence of marginal-stunting, wasting, obesity, overweight/obesity, underweight/wasting, and stunting with overweight or obesity in these participants was 15.5%, 2.4%, 2.9%, 33.2%, 13.1%, and 6%, respectively. The general characteristics of children across the tertiles of UPFs are presented in Table 1. According to this table, the amount of total energy intake (*p*-value<0.001) and socioeconomic status (*p*-value = 0.001) were higher in the highest tertile of UPF intake compared with the lowest tertile.

Energy-adjusted dietary intakes of participants across the tertiles of UPF intake are presented in Table 2. Values of dietary intake were calculated using an ANCOVA test controlling for energy intake. The intake of fat, carbohydrate, fruits, SFA, iron, magnesium, zinc, potassium, calcium, phosphorus, vitamin B9, vitamin B12, vitamin D, vitamin E, non-dairy drinks, cakes, and industrial bread, dairy drinks, salty snacks, processed meats, and fast food, sauces, and sweets was significantly higher across the tertiles of UPF intake. No other significant differences were observed for daily food consumption across the tertiles of UPF intake.

Risk of overweight/obesity, underweight/wasting, and stunting across the tertiles of UPF intake reported as OR and 95% CI are presented in Table 3. According to this table, higher tertiles of UPF intake were not associated with wasting, obesity, overweight and obesity, underweight and wasting, marginal-stunting, or marginal-stunting with overweight and obesity in crude or adjusted models.

## 4. Discussion

This study evaluated the association between UPF intake and malnutrition in children of Tehran. We found no significant associations between the consumption of UPFs and odds of wasting, obesity, overweight/obesity, underweight/wasting, marginal-stunting, or marginal-stunting/overweight/obesity. To the best of our knowledge, this is the first study that has evaluated the association between UPF consumption and malnutrition in children from the Middle East, including measures of overweight, obesity, wasting, and stunting.

The consumption of UPFs has been rapidly increasing throughout the world [22]. According to a food guide, assessing the intake of UPFs through childhood is important [30]. Eating behaviors are established in early childhood and have been shown to persist into adulthood, which are associated with various health outcomes [31]. Therefore, the association between UPF intake and malnutrition among children was assessed.

The present study found no significant association between UPF intake and overweight or obesity. A study in Brazil had similar findings for the association between UPF consumption and obesity in children [32]. Similarly, a study in Iran found no significant association between unhealthy food consumption and the weight status of children [33]. In the present study, mean intakes for calories and fiber increase across the tertiles of UPFs in parallel. It is well established that dietary fiber intake can reduce the presence and risk of obesity [34]. Therefore, it seems fiber may partially modulate the effects of increasing energy intake on obesity. In contrast to our findings, some studies have suggested that an increased intake of UPFs was associated with higher levels of body fat [35–37]. A study in Brazilian children found that the intake of UPFs was associated with abdominal obesity [22]. In Sweden, a study in adults found that the consumption of UPFs increases the prevalence of obesity [38]. Furthermore, a narrative review concluded that there is a positive association between UPF consumption and obesity [13]. Finally, many studies have found a significant association between obesity and the consumption of unhealthy foods [39–41]. The percent contribution of UPF to the total daily caloric intake was 57.31% in our sample, which is lower than the percent contribution in developed countries (up to almost 80% of total caloric intake in the US and Canada [17]). These differences may partially explain the findings in the present study. However, the absence of an association between UPF consumption and obesity in the present study may also be due to the age of the children as the duration of consuming UPFs may not be extensive enough to influence weight status, as it has been observed also elsewhere [32]. However, high intakes of UPFs consistently throughout early childhood may influence weight status in later childhood and beyond [42].

In the present study, higher intakes of UPFs had no significant associations with malnutrition including underweight, wasting, and stunting. In contrast to our findings, studies have indicated that the consumption of unhealthy foods was associated with unfavorable growth [43] and increased odds of undernutrition [44]. In the present study, the findings for the total dietary intakes of participants across tertiles were interesting. Across the tertiles of UPF consumption, the intakes of SFA and fats were higher, but the intakes of most vitamins, nutrients, fiber, and fruits were also higher. This may suggest different eating habits and dietary patterns in our study population compared to other populations. Therefore, although UPFs are considered low-quality contributions to the diet [22] and contain lower levels of nutrients [13], no significant associations were observed for underweight, wasting, and stunting.

Although no significant relationships between UPFs and malnutrition were found in this study, a few potential mechanisms may explain the expected relationships between UPF intake, malnutrition, and obesity [38]. Potential mechanisms include having a high energy density, flavourful taste promoting intake [45, 46], an effect on eating behavior and energy homeostasis, and an appetite regulator leading to overnutrition and a higher risk of obesity [47, 48]. Finally, UPFs reduce energy consumption in the body because they contain low ﬁber and nutrient content and high amounts of simple carbohydrates [49].

The strengths of this study are the large sample size and the use of validated and reliable dietary intake collection methods and standardized anthropometric protocols [23]. Study findings should be interpreted with consideration of limitations. First, the study design is cross-sectional and cannot determine a causal relationship between UPFs and risk of malnutrition. Dietary patterns may differ due to ethnicity or cultural practices, meaning our findings may not be generalizable to children in other settings. Finally, due to the observational nature of the study, there is a possibility of unmeasured confounding.

## 5. Conclusion

There was no evidence of an association between higher intake of UPFs and risk of wasting, overweight/obesity, underweight/wasting, marginal-stunting, or marginal-stunting/overweight/obesity in 6-year-old children in Tehran. Future studies evaluating the longitudinal relationships using prospective study designs are needed to better understand these relationships and confirm the findings in the present study.

## Figures and Tables

**Table 1:** Participant characteristics across the tertiles of UPFs^a^.

Variables	Tertiles of UPFs			*p* value^b^
	T1 (*n*:262) (6.66to 95.28)	T2 (*n*:264) (95.88 to 164.98)	T3 (*n*:262) (165.09 to 643.21)	
Physical activity (MET/h)	9.50 (0.36)	9.47 (0.35)	9.50 (0.34)	0.96
Total energy (kcal/d)	926.90 (246.38)	1035.99 (264.89)	1081.18(241.83)	<0.001
Socioeconomic status	20.11 (25.16)	22.08 (23.11)	27.22 (26.11)	0.001
Weight (kg)	20.96 (2.48)	20.75 (2.38)	20.85 (2.17)	0.59
Height (cm)	113.80 (2.01)	113.68 (2.02)	113.78 (1.97)	0.93
BMI (kg/m^2^)	16.18 (1.92)	16.06) 1.85)	16.11 (1.73)	0.64
BMI-for-age *z*-score	0.39 (1.15)	0.33 (1.09)	0.37 (1.03)	0.87
Height-for-age *z*-score	−0.54 (0.42)	−0.57 (0.41)	−0.54 (0.44)	0.93
Middle-arm circumference (cm)	15.45 (0.47)	15.51 (0.61)	15.40 (0.51)	0.29

^a^: Data are reported as mean (SD).^b^: Obtained from one-way ANOVA test. UPFs: Ultra-processed foods.

**Table 2:** Energy-adjusted dietary intakes of participants across the tertiles of UPFs.

Dietary intakes^b^	Tertiles of UPFs			*p* value^a^
	T1 (*n*: 262)	T2 (*n*: 264)	T3 (*n*: 262)	
	(6.66to 95.28)	(95.88 to 164.98)	(165.09 to 643.21)	
Protein (g/d)	38.68 (3.24)	37.06 (3.17)	42.51 (3.21)	0.46
Fat (g/d)	28.61 (0.72)	32.43 (0.70)	40.44 (0.71)	<0.001
Carbohydrate (g/d)	144.87 (3.01)	147.24 (2.94)	168.79 (2.98)	<0.001
Fiber (g/d)	18.48 (0.52)	17.12 (0.51)	19.10 (0.52)	0.02
Vegetables	182.9 (5.74)	186.6 (5.61)	197.6 (5.69)	0.17
Fruits	178.1 (7.52)	178.8 (7.35)	206.8 (7.46)	0.009
Whole grains	20.57 (1.01)	21.03 (0.99)	23.35 (1.00)	0.11
SFA (mg/d)	18.18 (0.52)	21.63 (0.51)	27.68 (0.52)	<0.001
Iron (mg/d)	15.41 (0.21)	15.82 (0.21)	17.49 (0.21)	<0.001
Magnesium (mg/d)	257.89 (6.03)	263.77 (5.90)	307.19 (5.98)	<0.001
Zinc (mg/d)	7.86 (0.11)	8.47 (0.11)	9.01 (0.11)	<0.001
Potassium (mg/d)	3135.59 (77.77)	3216.07 (76.03)	3692.05 (77.11)	<0.001
Calcium (mg/d)	455.03 (9.67)	489.19 (9.46)	551.39 (9.59)	<0.001
Phosphorus (mg/d)	558.65 (13.17)	613.05 (12.87)	738.01 (13.06)	<0.001
Vitamin B9 (*µ* g/d)	303.43 (9.91)	306.92 (9.69)	357.15 (9.83)	<0.001
Vitamin B12 (*µ*g/d)	3.34 (0.09)	3.80 (0.08)	4.18 (0.09)	<0.001
Vitamin A (RAE/d)	1356.66 (59.38)	1352.52 (58.06)	1420.83 (58.88)	0.65
Vitamin D (*µ* g/d)	1.10 (0.09)	1.70 (0.09)	2.13 (0.09)	<0.001
Vitamin K (*µ* g/d)	138.34 (5.38)	134.75 (5.26)	147.86 (5.33)	0.19
Vitamin E (mg/d)	10.16 (0.36)	12.27 (0.35)	12.45 (0.36)	<0.001
Non-dairy drinks (g/day)	2.43 (0.75)	6.27 (0.73)	13.21 (0.74)	<0.001
Cakes and industrial breads (g/day)	21.11 (1.72)	37.23 (1.68)	67.12 (1.71)	<0.001
Dairy drinks (g/day)	15.48 (2.51)	31.87 (2.46)	74.28 (2.49)	<0.001
Salty snacks (g/day)	3.91 (0.88)	8.26 (0.86)	17.55 (0.87)	<0.001
Processed meats and fast food (g/day)	10.99 (1.09)	19.85 (1.06)	34.13 (1.08)	<0.001
Sauces (g/day)	2.61 (0.40)	5.73 (0.39)	8.78 (0.40)	<0.001
Sweets (g/day)	9.67 (1.04)	17.21 (1.02)	24.90 (1.04)	<0.001

^a^: Calculated using univariate analysis of covariance. ^b^:All variables were adjusted for energy intake. *Note.* Values are reported as mean ± SE. UPFs: Ultraprocessed foods.

**Table 3:** Crude and adjusted multivariable OR and 95% CI for overweight/obesity, underweight/wasting, and marginal-stunting/overweight/obesity across the tertiles of UPFs.

Outcomes	Tertiles of UPFs			*P*-trend^a^
	T1 (*n*: 262) (6.66to 95.28)	T2 (*n*: 264) (95.88 to 164.98)	T3 (*n*: 262) (165.09 to 643.21)	
Wasting				
Crude^b^	1	0.61 (0.19–1.89)^c^	0.74 (0.25–2.17)	0.57
Model^d^	1	0.66 (0.21–2.09)	0.94 (0.30–2.87)	0.87
Obesity				
Crude^b^	1	1.43 (0.53–3.82)	0.85 (0.28–2.57)	0.79
Model^d^	1	1.57 (0.57–4.26)	0.97 (0.31–3.01)	0.98
Overweight/obesity				
Crude^b^	1	0.82 (0.57–1.17)	0.85 (0.59–1.23)	0.40
Model^d^	1	0.82 (0.56–1.18)	0.86 (0.59–1.25)	0.45
Underweight/wasting				
Crude^b^	1	0.96 (0.58–1.57)	0.78 (0.47–1.31)	0.36
Model^d^	1	0.89 (0.53–1.45)	0.69 (0.40–1.17)	0.17
Marginal stunting				
Crude^b^	1	1.05 (0.65–1.70)	1.19 (0.74–1.90)	0.46
Model^d^	1	1.03 (0.63–1.67)	1.16 (0.71–1.89)	0.53
Marginal stunting/overweight/obesity^*∗*^				
Crude^b^	1	0.66 (0.30–1.46)	1.27 (0.64–2.51)	0.46
Model^d^	1	0.67 (0.30–1.51)	1.25 (0.62–2.54)	0.47

^a^: Calculated using logistic regression. ^b^:Crude: Not adjusted for any variables. ^c^:Odds ratio (95% confidence interval).^d^: Model adjusted for energy intake, socioeconomic status, and physical activity. Obesity (BMI-for-age≥2SD), overweight (BMI-for-age≥1SD), underweight (BMI-for-age < −1SD), wasting (BMI-for-age < −2SD), marginal-stunting height-for-age *Z* (HAZ) scores were −2< HAZ ≤ −1. UPFs: Ultraprocessed foods. ^*∗*^: Marginal stunting with overweight or obesity.

## Data Availability

The datasets used and/or analyzed during the current study are available from the corresponding author on reasonable request.

## Abbreviations

UPFs: Ultraprocessed foods
OR: Odds ratio
CI: Confidence interval
BMI: Body mass index
FFQ: Food frequency questionnaire
SD: standard deviation
WHO: World Health Organization
SFA: Saturated fatty acid
*ANCOVA*: Analysis of covariance
PA: Physical activity.

## Supplementary Materials

Supplementary Materials Supplementary Table 1: NOVA classification and its groups. Supplementary Table 2: Food and beverage product items included as ultraprocessed foods in the present study.
