# Supplementary material for: Association between Ultraprocessed Food Intake and Overweight, Obesity, and Malnutrition among Children in Tehran, Iran
Source: Int J Clin Pract. 2022 Aug 24;2022:8310260. doi: 10.1155/2022/8310260 (PMC9433238; doi:10.1155/2022/8310260)
Supplement: Supplementary Materials — Supplementary Table 1: NOVA classification and its groups. Supplementary Table 2: Food and beverage product items included as ultraprocessed foods in the present study. [file 8310260.f1.docx]

**Supplementary Table 1**: NOVA classification and its groups.

| **NOVA Groups** | | **Components** |
| --- | --- | --- |
| **Group 1** | Unprocessed or minimally processed foods | Unprocessed (or natural) foods are edible parts of plants (seeds, fruits, leaves, stems, roots) or of animals (muscle, offal, eggs, milk), and fungi, algae, and water, after separation from nature. Minimally processed foods are natural foods altered by processes that include removal of inedible or unwanted parts, drying, crushing, grinding, fractioning, filtering, roasting, boiling, non-alcoholic fermentation, pasteurization, refrigeration, chilling, freezing, placing in containers, and vacuum-packaging. These processes are designed to preserve natural foods, to make them suitable for storage, or to make them safe or edible or more pleasant to consume. Many unprocessed or minimally processed foods are prepared and cooked at home or in restaurant kitchens in combination with processed culinary ingredients as dishes or meals |
| **Group 2** | Processed culinary ingredients | Processed culinary ingredients, such as oils, butter, sugar, and salt, are substances derived from Group 1 foods or from nature by processes that include pressing, refining, grinding, milling, and drying. The purpose of such processes is to make durable products that are suitable for use in home and restaurant kitchens to prepare, season, and cook Group 1 foods and to make with them varied and enjoyable hand-made dishes and meals, such as stews, soups, and broths, salads, breads, preserves, drinks and desserts. They are not meant to be consumed by themselves and are normally used in combination with Group 1 foods to make freshly prepared drinks, dishes and meals |
| **Group 3** | Processed foods | Processed foods, such as bottled vegetables, canned fish, fruits in syrup, cheeses and freshly made breads, are made essentially by adding salt, oil, sugar or other substances from Group 2 to Group 1 foods. Processes include various preservation or cooking methods, and, in the case of breads and cheese, non-alcoholic fermentation. Most processed foods have two or three ingredients and are recognizable as modified versions of Group 1 foods. They are edible by themselves or, more usually, in combination with other foods. The purpose of processing here is to increase the durability of Group 1 foods, or to modify or enhance their sensory qualities |
| **Group 4** | Ultra-processed foods | Ultra-processed foods, such as soft drinks, sweet or savoury packaged snacks, reconstituted meat products and pre-prepared frozen dishes, are not modified foods but formulations made mostly or entirely from substances derived from foods and additives, with little if any intact Group 1 food. Ingredients of these formulations usually include those also used in processed foods, such as sugars, oils, fats or salt. But ultra-processed products also include other sources of energy and nutrients not normally used in culinary preparations. Some of these are directly extracted from foods, such as casein, lactose, whey and gluten. Many are derived from further processing of food constituents, such as hydrogenated or interesterified oils, hydrolysed proteins, soya protein isolate, maltodextrin, invert sugar and high-fructose corn syrup. Additives in ultra-processed foods include some also used in processed foods, such as preservatives, antioxidants and stabilizers. Classes of additives found only in ultra-processed products include those used to imitate or enhance the sensory qualities of foods or to disguise unpalatable aspects of the final product. These additives include dyes and other colours, colour stabilizers; flavours, flavour enhancers, non-sugar sweeteners; and processing aids such as carbonating, firming, bulking and anti-bulking, de-foaming, anti-caking and glazing agents, emulsifiers, sequestrants and humectants. A multitude of sequences of processes is used to combine the usually many ingredients and to create the final product (hence ‘ultraprocessed’). The processes include several with no domestic equivalents, such as hydrogenation and hydrolysation, extrusion and moulding, and pre-processing for frying. The overall purpose of ultra-processing is to create branded, convenient (durable, ready to consume), attractive (hyper-palatable) and highly profitable (low-cost ingredients) food products designed to displace all other food group |

Taken from:

1. Monteiro CA, Cannon G, Moubarac JC, Levy RB, Louzada MLC, Jaime PC. The UN Decade of Nutrition, the NOVA food classification, and the trouble with ultra-processing. Public Health Nutr. 2018;21(1):5-17.

Schulze, Kai. Ultra-processed foods and cardiometabolic health. Diss. University of Cambridge, 2020.

**Supplementary Table 2**: Food and beverage product items included as ultra-processed food in the present study

| Food groups | Food and beverage items |
| --- | --- |
| Non-dairy Beverages | soft drinks |
| Industrial bread and cakes | baguettes, toasts, biscuits, Yazdi cakes, homemade cakes, other cakes, cookie, creamy sweet, and donuts |
| Dairy beverages | cocoa milk, chocolate milk, traditional ice cream, non-traditional ice cream |
| Potato chips and salty snacks | Crackers, Puffs, Chips |
| Processed meat and fast food | tuna, hamburgers, sausages, bologna, pizza |
| Suasues | ketchup, mayonnaise |
| Sweets | jam, Gaz, candy, Sohan, chocolate, caramel cream, rock candy, sesame halva, Nogal |

*Definition of ultra-processed food items adapted from the NOVA classification retrieved from Schulze, K., Ultra-processed foods and cardiometabolic health. 2020, University of Cambridge^24^.
